# Bacteriology

**Published:** 1905-12-23

**Authors:** 


### Progress in Medicine and Surgery.

### BACTERIOLOGY.

**Syphilis.**—Schaudinn and Hoffmann [1] describe a special spirochæte in the juice of syphilitic lymph glands. The organisms may be demonstrated in fresh or stained specimens on the surface or in the substance of syphilitic papules, primary sores, or indurated lymph glands. The stain used was azure blue and eosin. Two forms were found, one large and deeply staining, the other slender and faintly staining. It is to the latter that especial significance is attached. Material for examination is obtained by puncturing the glands with a hollow needle and drawing off the fluid. This slender organism, the Spirochæte pallida, was present in each of twenty-six cases examined. It is present in the inguinal glands before the secondary throat symptoms or rash appear. The organism is motile, long, thread-like, spiral, and has pointed extremities. It measures from 4 to 14$\mu$ in length and never exceeds .25$\mu$ in breadth. Each spirochæte presents from six to fourteen curves and these are sharp and well-marked. Metchnikoff and Roux,[2] having been presented with some of Schaudinn's preparations, proceeded to look for the spirillum in the lesions of apes artificially inoculated with syphilis, and obtained positive results in four out of six cases. No method of cultivating the spirochæte has yet been discovered and the organism has not been met with in other than syphilitic lesions. It is present in the lesions of congenital syphilis. Further particulars of the germs are furnished by McWerney [3] who found the spirochæte in every one of nine cases of primary and secondary syphilis. Smears were taken, fixed by immersion in absolute alcohol for ten minutes, stained for eighteen to twenty-four hours in Giemsa stain, and examined with a $\frac{1}{20}$in objective. Giemsa stain consists of Giemsa's azur I., azur II. eosin, dissolved in methyl alcohol and neutral glycerine, three drops of the compound to the c.cm. of water. A little of the material from a mucous tubercle put on in sterile broth as a hanging drop will serve to demonstrate the actively moving spirochæte. McWerney suggests that the spirillum has a nucleus or blepharoblast, an undulating membrane, and a substance largely composed of chromatin. The spirochæte is extra-cellular, but it is common to find organisms with one or both of their ends attached to a cell.

**B. Coli.**—Moorhead,[4] writing on the Bacillus coli as a cause of septicæmia gives a summary of cases hitherto reported. Escherich, who first described the organism, regarded it as a nonpathogenic form engaged in furthering the ultimate processes of digestion. Later, Javel, Wurtz, Martin, and Carega showed that the organism could excite suppuration, or play a subsidiary part in various toxic conditions, amongst which may be mentioned inflammatory intestinal lesions, peritonitis, cholangitis, cystitis, meningitis, pancreatitis, and arthritis. Cases of B. coli septicæmia, in which the organism has been isolated from the blood, are not common, but such have been reported by Henschen, Siredey, Moro, and Sittmar. Post-mortem examination often reveals a widespread dissemination of the colon bacillus, but many believe that the invasion takes place either immediately before or immediately after death. On the whole, however, it seems probable that if an organism is found in pure culture in many widely separated organs soon after death, then the infection was an ante-mortem one. The bacillus coli is capable of producing septicæmia in animals, and it has been the only organism found at post-mortem examination in fatal cases of septicæmia in man. Certain infectious diseases in animals are caused by B. coli and this bacillus has a powerful effect in modifying other forms of septicæmia, generally intensifying the disease. Moorhead gives in detail a case of coli blood infection observed by him in which the bacillus was found in many of the organs after death. Unfortunately all attempts to separate the colon bacillus from the blood during life were unsuccessful.

**The Smaller forms of Bacteria.**—The smallest known micro-organism [5] is that of fowl cholera measuring .3$\mu$. Yet it has been known from the days of Pasteur that varieties exist which are too small to be detected with the highest powers of the microscope. These bacteria are known by the cloudiness they impart to a fluid medium, by their passage through porcelain bougies, and by the pathological conditions which they excite. It is probable that their size is not much less than that of the smaller cocci. New staining methods, or more perfect methods of microphotography must be relied upon for their discovery. Sudertopf and Zsigmondy, by abolishing transmitted light and viewing the objects by means of rays of light obliquely directed, have made visible objects of a diameter of .05$\mu$. By this new means of illumination the germ of the disease pleuropneumonia of cattle has been demonstrated. A very fine grained chamberland candle will filter off even these minute germs, and they are readily destroyed by a temperature of 48°-56° F. Many of the diseases of animals are caused by these invisible germs, aphthous fever in cows, "horse-sickness," avian and bovine plague, and sheep-rot. Reed and Carrol have shown that the organism of yellow fever is capable of passing through a porcelain filter and is probably one of the minute bacteria. It is probable, too, that such diseases as variola, scarlatina, and rabies have a similar causation.

[1] Deut. Med. Woch., May 4, 1905. [2] Brit. Med. Journ., May 27, 1905. [3] Ibid., June 10, 1905. [4] Practitioner, June 1, 1905. [5] Med. Chron., Feb., 1905.

